# Characterization of Hspb8 in Zebrafish

**DOI:** 10.3390/cells9061562

**Published:** 2020-06-26

**Authors:** Magda Dubińska-Magiera, Joanna Niedbalska-Tarnowska, Marta Migocka-Patrzałek, Ewelina Posyniak, Małgorzata Daczewska

**Affiliations:** 1 Department of Animal Developmental Biology, Faculty of Biological Sciences, University of Wroclaw, Sienkiewicza 21, 50-335 Wroclaw, Poland; joannaniedbalska@gmail.com (J.N.-T.); marta.migocka-patrzalek@uwr.edu.pl (M.M.-P.); ewelina.posyniak@uwr.edu.pl (E.P.); 2 Hirszfeld Institute of Immunology and Experimental Therapy, the Polish Academy of Sciences, Rudolfa Weigla 12, 53-114 Wroclaw, Poland

**Keywords:** Hspb8, Bag3, Hspb1, muscle, zebrafish, autophagy, sHSP

## Abstract

Hspb8 is a member of the small heat shock protein (sHSP) family. Its expression is known to be upregulated under heat shock. This protein interacts with different partners and can, therefore, be involved in various processes relevant to tissue integrity and functioning. In humans, mutations in the gene encoding Hspb8 can lead to the development of various diseases such as myopathies and neuropathies. In our study, we aimed to perform an in-depth characterization of zebrafish Hspb8 during zebrafish development. We applied techniques such as RT-qPCR, Western blot, immunofluorescence, co-immunoprecipitation, LC-MS, and morpholino-mediated knockdown. We broadened the knowledge regarding zebrafish *hspb8* expression during development under normal and heat shock conditions as well as its tissue- and subcellular-specific localization. A co-IP analysis allowed us to conclude that zebrafish Hspb8 can interact with proteins such as Bag3 and Hsc70, which are crucial for formation of an autophagy-inducing complex. We also demonstrated that *hspb8* morpholino-mediated knockdown has an impact on zebrafish embryos’ morphology, muscle ultrastructure, and motility behavior. Our research provides a valuable resource for the potential use of the zebrafish as a model for studying pathological conditions associated with *hspb8* disorders.

## 1. Introduction 

Small heat shock proteins (sHSPs; HSPBs) belong to the extensive HSP family. They were named according to their small molecular weight within the range of 15–40 kDa. These chaperone proteins are ubiquitously expressed in different tissues and are present in both vertebrates and invertebrates. Their distinctive structural feature is the α-crystallin domain, which is crucial for the homodimer and heterodimer formation. The sHSPs are also able to form larger dynamic oligomers, which is necessary for regulation of their function [1]. The sHSPs can interact with different members of their own family, e.g., Hspb1 can bind to Hspb8 [2]. They also interact with a plethora of nonrelated protein partners, which makes them important factors in various cellular processes such as autophagy as well as during organism development [3,4].

At the molecular level, their most prominent task is to prevent the irreversible aggregation of non-native polypeptides, which may appear in the cell under various stress and pathological conditions. Unlike other chaperones, they perform their functions in an ATP-independent manner [1].

One of the reasons why sHSPs are in the center of interest of many researchers is the fact that mutations in the genes encoding them can be the cause of various human diseases such as myopathies and neuropathies [5,6,7,8].

Hspb8 (also known as Hsp22), a member of the sHSP family, is a protein commonly found in various organisms. It is expressed in muscle and nervous tissues, among others. Its expression is known to be upregulated under heat or toxic stress. Like any other members of the sHSP family, this protein interacts with different partners, and can, therefore, be involved in various processes relevant to tissue integrity and functioning.

It is well documented, thanks to the different studies carried out on various models such as human cell lines, that Hspb8 is involved in a specific form of autophagy, called chaperone-assisted selective autophagy (CASA) [9,10]. Interactions between Hspb8 and other components of the CASA, co-chaperone Bag3 (Bcl2-associated athanogene 3), Hsp70 (heat shock protein 70), and CHIP (carboxyl terminus of HSC70-interacting protein), act to deliver misfolded proteins to autophagosomes, which leads to their subsequent removal [9,11,12,13]. Proper functioning of the Hspb8/Bag3 complex is known to be crucial for skeletal muscle maintenance [14]. Of note, other sHSPs such Hspb1, a partner of Hspb8, may interact with this co-chaperone too [15,16]. 

As befits a multi-functional protein, Hspb8 is also involved in other crucial cellular processes, e.g., those connected with the dynamics of mitotic spindle orientation and cytokinesis [17,18]. Furthermore, its involvement in mitochondrial membrane potential maintenance and oxidative phosphorylation in mitochondria has also been confirmed [19,20]. 

Among the many proteins interacting with Hspb8 is RNA helicase Dd×20 (DEAD box protein Dd×20, gemin3, DP103), which itself binds to the SMN protein (survival of motor neuron) [21,22] and seems to be necessary during the early stages of embryogenesis [23]. 

As with other sHSPs, mutations in the *hspb8* gene can cause diseases included in the group of inherited motor neuron diseases (MNDs) such as distal hereditary motor neuropathy (dHMN type IIA) [24] and Charcot-Marie-Tooth disease type 2L (CMT2L) [25]. Specific mutations can also lead to the development of myopathies, among which distal myopathy [8,26] and rimmed vacuolar myopathy (RVM) [27] can be distinguished. 

In general, disease-associated mutation of *hspb8* or its functional counterparts can lead to loss of muscle and motor neuron integrity [4,28]. These findings are in line with other research that shows the depletion of other proteins involved in CASA, such as Bag3, can lead to the development of myopathic or neuropathic conditions [29,30].

Despite extensive studies undertaken by various research teams, the exact mechanisms underlying the development of different neuromuscular pathologies connected with a mutation in the gene coding for Hspb8 remain elusive. As alluded to earlier, there are many indications that the development of the mentioned diseases may be caused by the disruption of Hspb8 function in chaperone-associated autophagy, which is crucial for protection against neurotoxicity and the maintenance of skeletal muscle integrity.

The multitude of binding partners and processes in which Hspb8 is or may be involved in means that its role in muscle and nerve functioning and development is still not sufficiently explained. The elucidation of the relationship between the different mutations in the Hspb8 coding gene and the development of the neuropathic and myopathic phenotypes is also pending. Hence, there is a great need to look for new and efficient models that will help in answering these questions. 

So far, various transgenic mouse models of mutant *hspb8* have been established [31,32,33,34,35]. Discrepancies between observations made based on different transgenic models and human patients can be noted. Different models show a distinct spectrum of symptoms depending on the *hspb8* mutation type [8,19,35]. We still lack a detailed and complete explanation of the mechanism underlying the development of the disorders they cause. One should be aware that, because Hspb8 acts in complexes with other chaperones and partners, the consequences of the effects of the reduction of Hspb8 expression are difficult to predict.

In our study, we present the results of the analyses of Hspb8 expression level, its distribution, and its protein partner in zebrafish (*Danio rerio*). Since Hspb8, through the CASA complex, participates in myofibril stabilization [36], we wanted to check whether the zebrafish ortholog can interact with proteins such as Bag3 and Hsc70, which are crucial for forming the protein complex involved in this process. We also demonstrate the effects of *hspb8* morpholino-mediated knockdown on zebrafish embryos’ morphology, muscle ultrastructure, and motility behavior. We decided to conduct our study using this model organism since it offers several advantages such as the production of a large number of externally developing, transparent embryos, relatively short life cycle, and less expensive husbandry than mice. Moreover, the zebrafish proves to be quite a useful model to study muscle pathology, and it shares a high level of conservation (84%) of genes associated with human diseases [37,38]. Our choice of a model organism was also dictated by the fact that the role of sHSPs, including Hspb8, in muscle development and functioning in the zebrafish is relatively poorly understood. Our research provides valuable insights for the potential use of the zebrafish as a model for studying pathological conditions associated with *hspb8* disorders.

## 2. Materials and Methods

### 2.1. Ethical Statement

All experiments were carried out following ethical permits approved by the Local Ethics Commission in Wroclaw (108/2014), Poland.

### 2.2. Animal Maintenance and Handling

Zebrafish (*Danio rerio*), wild type strains (AB-Tu and Tubingen), were raised, staged, and maintained according to standard procedures [39,40]. The embryos were obtained by natural spawning and raised at 28 °C with a photoperiod of 14 h light/10 h dark. Zebrafish embryos were anesthetized by 0.04% tricaine in fish water in all experiments.

### 2.3. Heat Shock Assay

The 24-, 48-, 72-, 96-, and 120-h zebrafish embryos and larvae were collected in 50 mL Falcone tubes filled with preheated fish water and incubated for 1 h at 37 °C. After incubation, embryos were rapidly cooled to 28.5 °C and left for recovery for 1 h. The embryos were used for further analyses. 

### 2.4. RNA Isolation, Reverse Transcription, and Real-Time Quantitative PCR (RT-qPCR)

Total RNA from embryos was extracted using the Extracol reagent (EURX, Poland), following the provided protocol. RNA was quantified using the NanoDrop OneC Spectrophotometer (Thermo Scientific, Waltham, MA, USA). RNA integrity was confirmed by electrophoresis. The cDNA was synthesized using a High Capacity cDNA Reverse Transcription Kit (Applied Biosystems, MA, USA). Real-time quantitative PCR (RT-qPCR) was performed using the CFX Connect Real-Time PCR Detection System (Bio-Rad, CA, USA) utilizing the PowerUp SYBR Green Master Mix (Thermo Scientific, Waltham, MA, USA) with the gene-specific primers indicated below (Table 1.).

The software automatically determined the Ct values. Standard curves for each pair of primers were prepared by serial 5-fold dilutions of the template cDNA followed by the determination of reaction efficiencies.

### 2.5. In Situ Hybridization

The 48- and 60-h zebrafish embryos after heat shock assay were collected and fixed in fresh 4% paraformaldehyde, dehydrated in methanol, and proceed using the standard whole-mount in situ hybridization (WISH) procedure [41]. In situ probe primers targeting hspb8 (accession number NM_001100957.2) were synthesized using forward (5′–CAAGCCCGAAGAGCTTA-3′) and reverse (5′–GACTTCAACCACAACCTTTGA -3′) primers. All probes were amplified from hspb8 cDNA in the pCMV-SPORT6.1 vector (GeneBank CD282221.1). Then, in situ probe synthesis was performed using Digoxigenin RNA Labeling Kit (SP6/T7, Roche Diagnostics, Mannheim, Germany). Embryos were mounted into low melting agarose and imaged using a light microscope (Leica DM5000, Leica, Munich, Germany).

### 2.6. SDS-PAGE and Western Blot

Protein samples for SDS-PAGE and Western blot (WB) analysis were prepared as follows. Manually dechorionated zebrafish embryos were anesthetized and homogenized using a plastic pestle in the extracting buffer, which was simultaneously the gel loading buffer (GLB) (120 mM Tris-HCl pH 6.8; 2% SDS; 2 mM DTT; 20% glycerol; 5% 2-mercaptoethanol; 0.01% bromophenol blue). The use of this buffer allowed for the most effective isolation of Hspb8 protein from embryos compared to other standard lysis buffers such as RIPA lysis buffer (data not shown). The limitation of this approach is the inability to assess the amount of protein in the sample using biochemical methods, such as the BCA or Bradford method. Therefore, when preparing lysates, special attention was paid to maintaining the constant proportions of lysis buffer in relation to the body volume of the embryo (1µl of GLB per 1 U of the embryo body volume). The embryos’ volume was assessed by multiplications of the body length and width, which was measured just above the yolk. The measurements were performed using ImageJ software [42] using photos of the individual embryo obtained with the light microscope (Leica DM5000, Leica, Munich Germany). The multiplication of these dimensions equal to 1 mm^2^ was considered as one unit (U). 

Equal volumes of prepared samples were used for electrophoresis. Proteins were separated using 10% (for zebrafish embryo lysates) and 12% (for co-immunoprecipitation eluates) SDS-PAGE and electrotransferred onto 0.2 µm nitrocellulose or 0.42 µm PVDF membrane (GE Amersham, IL, USA). For determination of the molecular mass, a protein ladder was used (Perfect Tricolor Protein Ladder, EURX, Poland). Immunodetection was performed using primary anti-Hspb8 (Thermo Scientific, Waltham, MA, USA) and/or secondary anti-β-actin-HRP and goat anti-rabbit IgG HRP-conjugated antibodies (Santa Cruz Biotechnology, TX, USA) in the ChemiDoc XRS + (Bio-Rad, CA, USA) or G-Box (Syngene, MD, USA) imaging systems. To control the specificity of commercially available antibody raised against human Hspb8 (Thermo Scientific, Waltham, MA, USA), Western blot analysis was performed with the use of the recombinant zebrafish Hspb8 (Figure S2).

### 2.7. Co-Immunoprecipitation Assay

Protein samples for the co-immunoprecipitation (co-IP) assay were prepared as follows. The 120 hpf (hours post fertilization) zebrafish larvae were heat shocked, anesthetized, and homogenized on ice in an Eppendorf tube containing 200 μL of lysis buffer from the Pierce Co-Immunoprecipitation (Co-IP) Kit (Thermo Scientific, Waltham, MA, USA) using a plastic pestle. To prevent protein degradation, the lysis buffer was enriched with 0.1 mM phenylmethane sulphonyl fluoride (PMSF) and 10 μL/mL inhibitor cocktail (Sigma-Aldrich, St. Louis, MO, USA). The fresh homogenate of 30 individuals, containing 480 mg protein, was used for each co-IP experiment. Lysates were incubated on ice for 30 min before being centrifuged (13,000× *g* RCF, 15 min, 4 °C). The supernatants were collected and immediately used for performing co-IP according to the manufacturer’s protocol.

Three co-IP columns, containing an amine-reactive resin that covalently couples antibodies, were used in each experiment: one containing resin with the immobilized anti-Hspb8 antibody (Thermo Scientific, Waltham, MA, USA), one with the anti-Bag3 antibody (Abcam, Cambridge, UK), and one with resin only (negative control). The obtained eluates were analyzed via SDS-PAGE and, following Western blot, liquid chromatography-mass spectrometry (LC-MS).

### 2.8. LC-MS Analysis

Protein samples (co-IP eluates) were homogenized and prepared as described by Orlowska et al. (2013) [43]. Proteins were separated by SDS-PAGE and stained by the modified Coomassie staining method. Fragments of the gel were cut out (size of cutouts corresponded to the size of bands that had been identified as a protein of interest using the Western blot technique). Samples were sent for identification by LC-MS to the Mass Spectrometry Laboratory, IBB PAS, Warsaw, Poland. Peptides were analyzed by LC-MS-MS/MS (liquid chromatography coupled to tandem mass spectrometry) using the Nano-Acquity (Waters) UPLC system and QExative Orbitrap mass spectrometer (Thermo Fisher Scientific) and by applying peptides to the precolumn (nanoACQUITY UPLC Trapping Column Waters) using water containing 0.1% formic acid as a mobile phase. Then, the peptides were transferred to a nano-column (nanoACQUITY UPLC BEH C18 Column (75 μm inner diameter; 250 mm long; Waters)) using an acetonitrile gradient (5–35% AcN in 70 min) in the presence of 0.1% formic acid with the flow rate of 250 nL/min. Three washing runs ensuring a lack of cross contamination from previous samples preceded each analysis. The column outlet was directly coupled to the ion source of the spectrometer working in the regime of a data-dependent MS to MS/MS switch. Peptides were eluted directly to the ion source of the mass spectrometer. Before each LC run, a blank run was performed to ensure no material was carried over from a previous analysis. Data were acquired in the m/z range of 300–2000. Data were searched using Mascot (Matrix Science) against the NCBI *Danio rerio* database (55 729 entries). The variable modification was oxidation (M), while the fixed modifications were carbamidomethyl (C), peptide mass tolerance 20 ppm, fragment ion tolerance 0.1 Da, and 1 missed cleavage.

### 2.9. MO Microinjection

The morpholino oligonucleotides (MOs) were synthesized by Gene Tools, LLC (Philomath, OR, USA) [44]. The morpholinos were designed to interfere with Hspb8 translation. Two different, non-overlapping MOs targeting the *hspb8* gene were used. The morpholino sequences used in this study and their concentrations are indicated below (Table 2.).

To determine the lowest effective dose, different concentrations of MOs were injected into zebrafish embryos [45]. Both MOs led to the same, altered phenotype. The MOs were diluted in phenol red solution (Sigma-Aldrich, St. Louis, MO, USA) and then heated for 10 min at 95 °C. The quantity of the MO was calibrated and injected into 1–2 cell stage zebrafish embryo. 

### 2.10. Phenotypic Analysis

Phenotypic analysis was conducted with a light microscope (Leica DM5000, Leica, Munich Germany). The 48 hpf zebrafish embryos were anesthetized, placed on a depression glass microscope slide, and photographed. Visual assessment was performed to calculate the percentage of animals with normal phenotype (15–20 individuals in each group). Observers, who were blinded to the morpholino treatment status of the zebrafish embryos, assessed morphant phenotypes. The embryos were classified into two categories: normal and disrupted (described as individuals with a curved body, altered tail region, and pericardial edema).

### 2.11. Birefringence Assay

Birefringence is a common non-invasive assay used to determine the degree of muscular disorganization of zebrafish embryos during early development. The 48 hpf zebrafish embryos were anesthetized and placed on a glass microscope slide. While the polarizing filters were crossed, the fish were rotated to find the angle that maximized birefringence. The microscope exposure was adjusted to see the light refracting through the striated muscle of the wild type, non-treated fish. All settings remained unchanged during the morphants’ examinations (15–20 animals in each group). The observations were performed, and images were acquired using the Leica DM5000 light microscope (Leica, Munich Germany) with a pair of polarized lenses. ImageJ software was used to quantify the birefringence [42,46].

### 2.12. Fluorescent Immunohistochemistry 

The zebrafish embryos were anesthetized and fixed in 4% paraformaldehyde (PFA) in phosphate buffer saline (PBS) for 45 min at room temperature. The samples were transferred to 30% sucrose in PBS for overnight incubation at 4 °C. Next, samples were embedded in the optimal cutting temperature medium (Tissue-Tek O.C.T, Sakura Finetek, CA, USA), placed in a cryomold, and frozen at −80 °C. The samples were cut into 14-μm sections in a cryostat (Leica, Munich Germany) at −24 °C, placed on SuperFrost Plus slides, and subjected to immunofluorescence staining. 

Samples were blocked with 1% bovine serum albumin (BSA) in PBST (PBS with 0.1% Tween-20) for one hour at room temperature. All of the wash steps were conducted with PBST. Incubation with primary antibodies was conducted overnight at 4 °C and with secondary antibodies for one hour at room temperature. The following primary antibodies were used: rabbit polyclonal anti-Hspb8 (Pierce, Thermo Scientific, Waltham, MA, USA) at dilution of 1:200 in PBST, mouse monoclonal anti-α-actinin (Sigma-Aldrich, St. Louis, MO, USA) at dilution of 1:200 in PBST, mouse monoclonal anti-Myosin Heavy Chain, MYH1, F59 (Developmental Studies Hybridoma Bank, IA, USA) at dilution 1:50 in PBST. The following secondary antibodies were used: goat polyclonal anti-mouse IgG FITC conjugated (Sigma-Aldrich, St. Louis, MO, USA) at a dilution 1:50 in PBST, donkey polyclonal anti-rabbit IgG Cy5 conjugated (Jackson ImmunoResearch, West Grove, PA, USA) at dilution 1:100 in PBST, donkey polyclonal anti-mouse IgG Cy3 conjugated (Jackson ImmunoResearch, West Grove. PA, USA) at dilution 1:100 in PBST. Additionally, for F-actin identification, Alexa Fluor 546-conjugated phalloidin was used (Thermo Scientific, Waltham, MA, USA). The DNA was stained with 4,6-diamidino-2-phenylindole (DAPI; 0.2 μg/mL). The samples were mounted in a fluorescent mounting medium (Dako, Agilent, CA, USA). For the imaging, an Olympus Fluo View FV1000 confocal laser scanning microscope (Olympus, Japan) was used. Images were processed using an FV10-ASW_Viewer.

### 2.13. Transmission Electron Microscopy

For electron microscopic techniques, the 48 hpf zebrafish embryos were anesthetized and fixed with 2.5% glutaraldehyde (Sigma-Aldrich, St. Louis, MO, USA) in 0.1 M phosphate buffer, pH 7.4, for 24 h at 4 °C. The samples were repeatedly rinsed in the same buffer and post-fixed for 2 h in 1% OsO_4_ (Sigma-Aldrich, St. Louis, MO, USA) in 0.1 M phosphate buffer. In the next step, the material was dehydrated in a graded acetone series (30, 50, 70, 90, and 100% for 5 min each), embedded in a mixture of acetone-epoxy resin (Epon 812, Sigma-Aldrich, St. Louis, MO, USA) in a 1:1 ratio, and incubated for 24 h, 60 °C in a glass dish. The dish was opened during the last 7 h of incubation to let the acetone evaporate. Polymerization of the epoxy resin with embedded embryos was performed at 45 °C for 24 h, and then 60 °C for 72 h. The Epon blocks were cut on Leica Ultracut UCT (Leica, Munich Germany). Ultrathin sections were collected on 200 mesh copper grids, stained with uranyl acetate and lead citrate according to the standard protocol [47], and examined in a JEM-1011 electron microscope (JEOL, Tokyo, Japan).

### 2.14. Touch-Evoked Response Assay

To analyze the motility of the zebrafish embryos, a touch-evoked response (TER) assay was used [48]. Manually dechorionated 72 hpf embryos were placed in a 5 cm Petri dish filled with fish water and allowed to accommodate. The embryos were gently touched at the head with a needle, and the motion was recorded with a digital video camera at the frequency of 25 fps (frames per second). Files were converted into AVI format, and the movements of each embryo were manually assessed using ImageJ [42]. The motion trajectory of individuals was determined, and the distance was measured. Embryos that did not show any response to touch were excluded from further analyses. 

### 2.15. Statistical Analysis

Data concerning real-time quantitative PCR for *hspb8* expression level at different developmental stages before and after upregulation (heat shock), phenotype, birefringence analysis, and evaluation of morpholino-mediated knockdown efficiency are given as means ± standard deviations, and their significance was determined with Student’s *t*-test. 

Data regarding the touch-evoked response assay and real-time quantitative PCR were statistically analyzed using the ANOVA test followed by the Games–Howell post hoc test due to the lack of homogeneity of variance.

A significance level of *p* < 0.05 and *p* < 0.001 (indicated as * and **, respectively) was used in all statistical analyses. At least three independent experiments were carried out. 

The data analysis for this paper was generated using the Real Statistics Resource Pack software (Release 6.8), copyright (2013–2020) Charles Zaiontz.

## 3. Results

### 3.1. Zebrafish Hspb8 Expression, Localization, and Interactions

#### 3.1.1. Tissue-specific and Subcellular Localization of Hspb8 during Zebrafish Development under Normal and Heat Shock Conditions

Under stress conditions such as heat shock, the concentration of small heat shock proteins within the cell is increased. This allows their chaperone effectiveness to be increased, which involves binding to misfolded proteins and thereby avoiding the formation of aggregates [11,49]. Some studies concerning this issue in the case of zebrafish *hspb8* expression during fish development were previously performed [50,51].

**Figure 1:**
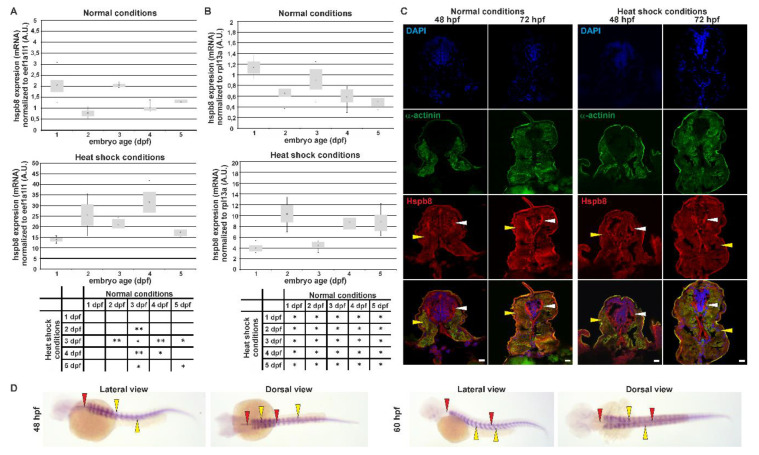
Tissue-specific expression and localization of Hspb8 during zebrafish development under normal and heat shock conditions. A and B. Real-time quantitative PCR (RT qPCR) of *hspb8* mRNA expression level during zebrafish development (from 1 to 5 dpf) in normal (upper part A and B) and heat shock conditions (bottom part A and B). Expression of *hspb8* mRNA was normalized to *eef1a1l1* (**A**) and *rpl13a* (**B**). Error bars show the standard deviation. The tables below indicate the pairwise comparison between *hspb8* expression level during zebrafish development (from 1 to 5 dpf) under normal and heat shock conditions. Statistically significant differences are indicated with *; * *p* < 0.05 and ** *p* < 0.001 (Student’s *t*-test). The experiment was performed 3 times, *n* = 25–30. A.U. = arbitrary unit. (**C**) Cross-sections of the mid-trunk region of 48 and 72 hpf zebrafish embryos under normal and heat shock conditions. The Hspb8 (red) localizes in muscle cells (yellow arrowheads) and the lateral spinal cord (white arrowheads). α-Actinin (green) is used to show muscles. Nuclei are stained blue. Cryosection (14 μm thick). Images represent single cross-sections of the mid-trunk region (0.45 μm thick). Scale bar: 10 μm. Images were obtained with a confocal laser scanning microscope (Olympus FV1000). (**D**) *hspb8* expression in 48 and 60 hpf zebrafish embryos under heat shock conditions, whole-mount in situ hybridization. The *hspb8* mRNA localizes in muscles (yellow arrowheads) and the lateral spinal cord (red arrowheads).

In our study, we extended previous studies. We analyzed *hspb8* expression at the mRNA level using real-time quantitative PCR from the first to the first day after fertilization at 24-h intervals (Figure 1A,B). The developmental expression patterns of *hspb8 ^eefa1l1^* and *hspb8 ^rpl13a^* at the mRNA level were similar. We observed statistically different *hspb8* expression levels at different developmental stages (days post fertilization, dpf) (F (4, 13) = 7.6, *p* = 0.002 for *hspb8 ^eefa1l1^*, and F (4, 13) = 3, 9, *p* = 0.027 for *hspb8 ^rpl13a^*). The highest *hspb8* levels were seen on the first and the third dpf.

In response to a one-hour heat shock at 37 °C, we found that the expression of *hspb8* was significantly upregulated in all analyzed developmental stages. The largest change was observed in 2 dpf and 4 dpf embryos, both in the *hspb8 ^eefa1l1^* (over 30-fold) and *hspb8 ^rpl13a^* (over-15 fold) level. In the case of *hspb8 ^rpl13a^,* an additional 17-fold increase in mRNA level was observed on 5 dpf. 

The age groups before and after heat shock were compared by Student’s *t*-test showing statistical differences for embryos (Figure 1A,B). In conclusion, *hspb8* was upregulated by heat shock during zebrafish development (from 1 to 5 dpf).

We also examined the Hspb8 localization within the developing zebrafish embryo both under normal and heat shock conditions (Figure 1C). Hspb8 was mostly expressed both in muscle and nervous tissues before and after heat shock. We observed no significant change in Hspb8 distribution at the tissue level after heat shock (Figure 1C). Our observations regarding Hspb8 tissue distribution made at the protein level are consistent with those at the mRNA level (Figure 1D).

**Figure 2:**
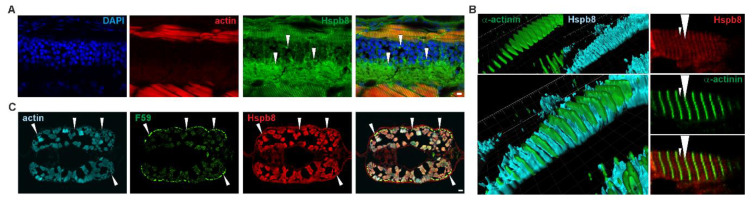
Tissue-specific and subcellular localization of Hspb8 during zebrafish development. (**A**) Hspb8 (green) subcellular distribution in the cytoplasm of neurons (white arrowhead) in the lateral spinal cord. Actin detected with Fluor 546-conjugated phalloidin (red). DNA was stained with DAPI (blue) in the lateral sections of the mid-trunk region of 72 hpf zebrafish embryos. Scale bar: 5 μm. (**B**) Hspb8 subcellular distribution in muscle fiber of 120 hpf zebrafish larvae. Left: 3D reconstruction (Imaris) of Hspb8 (light blue) and α-actinin (green, a marker of Z-line). Right: Localization of Hspb8 (red) and α-actinin (green) in muscle fiber of 120 hpf zebrafish larvae. Small arrowhead indicates M-line, large arrowhead indicates Z-line. Images were obtained with a confocal laser scanning microscope (Olympus FV1000). Cryosection (14 μm thick). Images represent the single Z-section (0.45 μm thick). (**C**) Hspb8 (red) is present in both white and slow muscles (white arrowhead). The slow muscle was detected using the F59 antibody (green) and actin with Fluor 546-conjugated phalloidin (light blue) in the cross-sections of the mid-trunk region of 120 hpf zebrafish embryo. Scale bar: 10 μm.

Immunofluorescence staining of developing zebrafish revealed that Hspb8 was expressed in nervous tissue within the cytoplasm of neurons in the lateral spinal cord (Figure 2A). Detailed analysis of Hspb8 localization revealed its presence in muscle cells in the proximity of Z and M lines (Figure 2B). Moreover, through the use of the F59 antibody, which allows identification of muscle fibers expressing the slow myosin heavy chain in early zebrafish embryos [52,53], we confirmed that Hspb8 localized in both slow and fast fibers (Figure 2C). 

#### 3.1.2. Protein Partners of Zebrafish Hspb8

**Figure 3:**
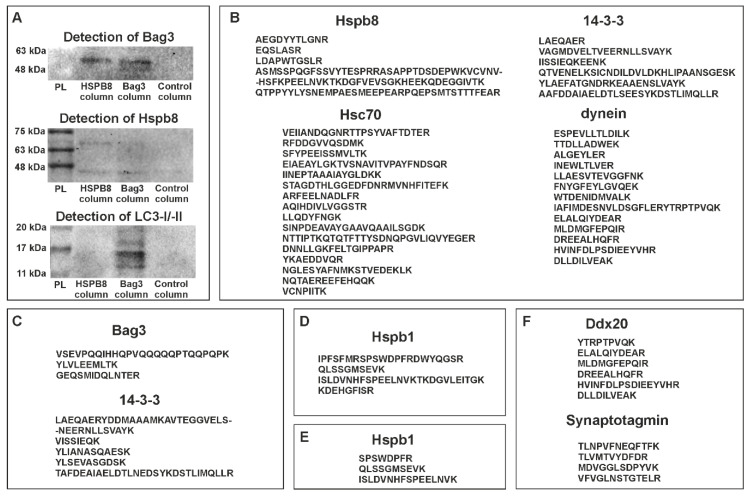
Protein partners of zebrafish Hspb8. (**A**) Western blot analysis of co-immunoprecipitation (co-IP) assay results. Each experiment involved the use of three columns: the first containing resin with an immobilized anti-Hspb8 antibody, the second an immobilized anti-Bag3 antibody, and the third only resin was used as a negative control. Hspb8 and Bag3 interact with each other. Moreover, Bag3 interacts with LC3 I/II. (**B**) and (**C**), List of peptides representing proteins involved in the formation of the autophagy-inducing complex detected by LC-MS analysis of eluates obtained from the co-IP experiment conducted using the column with an immobilized anti-Bag3 antibody (**B**), anti-Hspb8 antibody (**C**). (**D**) and (**E**), List of peptides representing Hspb1 detected by LC-MS analysis of eluates obtained from the co-IP experiment conducted using the column with an immobilized anti-Bag3 antibody (**D**) and anti-Hspb8 antibody (**E**). (**F**) List of peptides representing neuronal proteins detected by LC-MS analysis of eluates obtained from the co-IP experiment conducted using the column with an immobilized anti-Hspb8 antibody.

To obtain information enabling us to perform a more complete characterization of zebrafish Hspb8 and acquire data on its potential interactions with other proteins, we decided to carry out the co-immunoprecipitation (co-IP) assay. This assay assumes the use of target protein-specific antibodies to indirectly capture proteins that are bound to a specific target protein. We conducted two independent experiments that differed in the type of antibody used. In the first one, we used an anti-Hspb8, whereas the second one was based on the anti-Bag3 antibodies. The data were assessed using Western blot (Figure 3A) and mass spectrometry LC-MS (Figure 3B–F) analyses. The obtained data allowed us to conclude that similarly to other organisms, zebrafish Hspb8 can be involved in the formation of the autophagy-inducing complex. Western blot analysis provided proof for an interaction between Hspb8 and Bag3. Moreover, we also confirmed that Bag3, contrary to Hspb8, may interact with LC3 I/II, which is a central protein in autophagosome biogenesis (Figure 3A). Our results (except for LC3) were validated in the LC-MS method. As an outcome of the LC-MS analysis of eluates obtained from the co-IP experiment, proteins engaged in the formation of the autophagy-inducing complex such as Hsc70, dynein, and 14-3-3 protein were also detected (Figure 3B,C). Additionally, we confirmed that zebrafish Hspb8 and Bag3 can interact with Hspb1 (Figure 3D,E).

Although, when conducting this part of our study, we used lysates prepared from entire larvae (120 hpf), we suppose that interactions of Hspb8 with its partners display tissue-specific aspects. This assumption is based on the results of LC-MS analysis, which revealed the presence of tissue-specific proteins within samples obtained from the co-IP assay (Figure 3F). The mentioned tissue-specific context of zebrafish Hspb8 functioning relates to the development and survival of the neuronal cell. Data from the identification of peptides present in samples obtained from the co-IP assay based on the use of an anti-Hspb8 antibody indicate that there is a possibility of interaction between Hapb8 and Dd×20, which is the partner of the SMN protein (survival of motor neuron), and synaptotagmin (Figure 3D). This suggests Hspb8 involvement in neuronal cell functioning.

### 3.2. Effects of Morpholino-Mediated Knockdown of Zebrafish Hspb8 

#### 3.2.1. Morphological Analysis of Zebrafish Embryos with Decreased Hspb8 Level

Spatial-temporal expression of Hspb8 during zebrafish development suggests its involvement in muscle and nervous system maturation and functioning. To test whether zebrafish Hspb8 is important during zebrafish development, the gene coding for it was knocked down in zebrafish embryos by injecting translation-blocking morpholino oligonucleotides (MO). To control the specificity of the knockdown effects, we used two different morpholinos (M1, M2). The morpholinos were injected into 1- to 2-cell-stage zebrafish embryos. The effectiveness of the Hspb8 knockdown was assessed using Western blot analysis (Supplemental Figure S1). It was performed by comparison of the optical density of Hspb8 to beta actin bands. The results showed that the Hspb8 expression level was significantly lower in both groups of morphants (M1, M2) than in the control (NT, non-treated).

**Figure 4:**
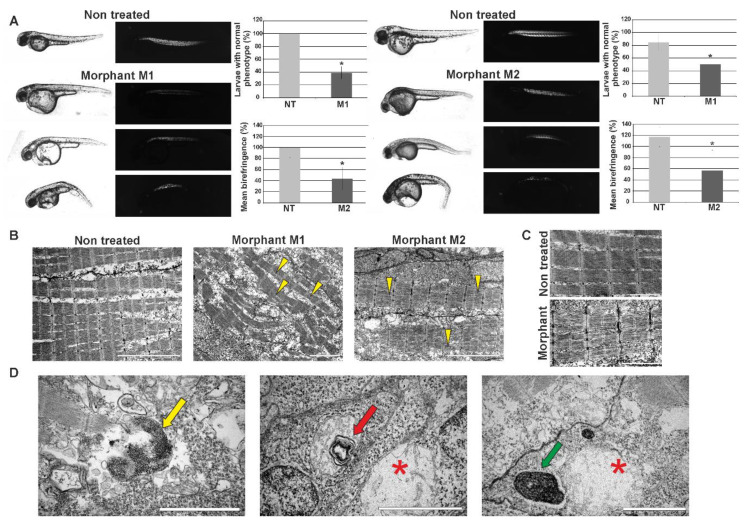
Effects of morpholino-mediated knockdown of zebrafish *hspb8* on zebrafish embryo morphology and muscle structure. The effect of *hspb8* knockdown was obtained through injections of morpholino oligonucleotides. 48 hpf zebrafish morphants (M1 and M2) were compared with control embryos (NT, non-treated). (**A**) The upper part of the panel shows representative images taken in normal light (the changes in morphology such as curved body, altered tail region, and pericardial edema are visible) and images presenting birefringence obtained in polarized light (Leica DM5000 light microscope, Leica, Munich Germany). The bottom part of the panel shows the phenotype quantification and its statistical evaluation. The phenotype quantification was conducted based on embryos morphology and their trunk muscle birefringence. The number of individuals in the control group (NT) with normal phenotype was taken as 100%. The analysis revealed that the differences between morphants (M1, M2) and control (NT) groups are statistically significant (indicated by an asterisk). The phenotype observed in over 50% of morphants was manifested in differences in body size and shape, particularly in the tail part. Moreover, pericardial edema, which is a consequence of abnormal accumulation of fluid in the pericardial cavity, occurred. The quantification of trunk muscle birefringence of individuals in the control group (NT) was taken as 100%. Statistical analyses were performed using Student’s *t*-test, *p* < 0.05, *n* = 20–15 in each group; each experiment was repeated at least three times. Asterisks (*) indicate significantly different groups. Error bars show the standard deviation. (**B**) Ultrastructural analysis of morphants’ (M1, M2) and control embryos’ (NT) muscle. Note the disruption of sarcomere organization in morphants’ muscles. The disruption is manifested as the appearance of gaps between filaments within the sarcomere (yellow arrowheads). Scale bar: 5 μm. (**C**) Magnified regions of sarcomeres in muscles of morphant and control individuals (non-treated). Note the disruption of sarcomere organization and accumulation of glycogen granules in morphants’ muscles. Scale bar: 2 μm. (**D**) Ultrastructural analysis of structures present in morphants’ muscles. The membrane-bound autophagosomes containing glycogen (yellow arrow); mitochondrion with mitochondrial vacuolization (red arrow) in the vicinity of the swollen mitochondrion (red asterisk); structure built of a whorl-like inner membrane (green arrow) in the vicinity of a swollen mitochondrion (red asterisk). Scale bar: 0.5 μm.

We assessed 48 hpf zebrafish embryo phenotypes using a light microscope (Figure 4A). The number of individuals in the control group (non-treated (NT)) with normal phenotype was taken as 100%. Morphants (M1, M2) showed abnormalities in morphology such as curved body, altered tail region, and pericardial edema (which is a consequence of an abnormal accumulation of fluid in the pericardial cavity). The abrupt phenotype was observed in 61% of M1 and 50% of M2 morphants (Figure 4A). 

Further phenotype assessment was conducted based on embryos’ trunk muscle birefringence observed in polarized light. The normal muscle structure is visible as bright birefringence. The birefringence of individuals in the control group (NT) was taken as 100%. Morpholino-mediated knockdown of *hspb8* in zebrafish embryos caused changes in muscle structure. These changes were manifested by an overall reduction of the birefringence of morphants’ muscles (to 43.4% in the case of M1 and 56.4% in the case of M2 morphants). The analysis of the results revealed that the differences between morphants and controls were statistically significant (Figure 4A).

To validate the birefringence observations that revealed the alteration in morphants’ skeletal muscle structure, we carried out ultrastructural analysis using a transmission electron microscope (Figure 4B). The conducted analysis revealed the disruption of sarcomere organization and the accumulation of glycogen granules in morphants’ muscles. The disruption was manifested as the appearance of gaps between filaments within the sarcomere (Figure 4B,C). We also observed in morphants’ muscles the presence of structures such as membrane-bound autophagosomes containing glycogen, a mitochondrion with mitochondrial vacuolization, a swollen mitochondrion, and a structure built of a whorl-like inner membrane (Figure 4D). 

Our observations may suggest that Hspb8 is involved in muscle development and/or the maintenance of muscle structure in zebrafish. 

#### 3.2.2. Decreased hspb8 Level Alters Swimming Behavior of Zebrafish Embryos

**Figure 5:**
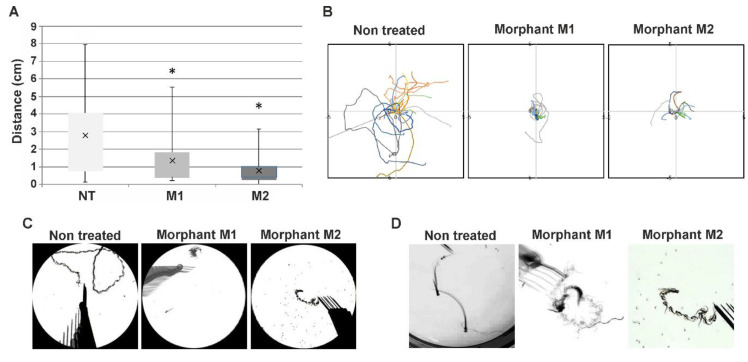
Altered swimming behavior in zebrafish embryos with decreased *hspb8* level. (**A**) Box plot represents distribution of swimming distances in the touch-evoked response (TER) assay, measured in cm; NT: non-treated embryos (*n* = 86); M1 (*n* = 28) and M2 (*n* = 75) morphants: embryos after *hspb8* knockdown using, respectively, morpholino oligonucleotides (MO) 1 and 2. Statistical analyses were performed using ANOVA followed by the Games–Howell post hoc test, *p* < 0.05; each experiment was repeated at least three times. Asterisks (*) indicate significantly different groups. Error bars show the standard deviation. (**B**) The colored lines demonstrate video recorded tracks of swim trajectories of individual embryos (*n* = 10) stimulated to move by a physical touch to the head. (**C**) Representative traces of individual swimming episodes of investigated embryos showing the typical (in the case of the non-treated group) and abrupt (in the case of M1 and M2 morphants) trajectories. (**D**) Magnified view of traces of individual swimming episodes of investigated embryos showing the typical (in the case of the non-treated group) and abrupt (in the case of M1 and M2 morphants) trajectories.

To assess the effect of the decrease of the Hspb8 level on zebrafish locomotor activity, we decided to conduct behavioral tests that evaluate zebrafish swimming abilities. The zebrafish embryo exhibits a well-developed escape response to touch at 72 hpf [48]. The response is characterized by rectilinear swimming away from the stimulus. A touch-evoked escape response (TER) assay was used to quantify swimming distance, as well as to assess the trajectory phenotype of morphant (M1 and M2) and control (NT, non-treated) groups (Figure 5). In the control group (NT), all embryos responded to touch, while in morpholino-injected groups, the number of responding individuals was 80% for the M1 and 92% for the M2 group, respectively. Embryos that did not respond were not further analyzed. 

The average distance that morpholino-injected embryos traveled was 1.35 cm for M1 and 0.76 cm for M2. In both cases, the distance was significantly shorter in comparison to the control group (average 2.8 cm) (Figure 5A). Additionally, we noted differences in the scheme of embryo movement in response to tactile stimuli. While control group embryos exhibited a rectilinear type of movement typical for this age, morpholino-injected embryos moved by turning around their axis in a sinusoidal manner (Figure 5B). The pattern of movement is reflected in the trajectory/traces graphs and images created from the merger of single film frames of individual embryos (Figure 5C,D). These results indicate that the knockdown of *hspb8* caused locomotion impairment both in distance traveled and the nature of movement in zebrafish embryos. 

## 4. Discussion

Increased expression of small heat shock proteins under various stress conditions such as heat shock allows them to conduct their chaperone activity [11,49]. In the case of Hspb8, its expression in zebrafish has already been the subject of research, although to a limited extent [50,51]. Previous studies concerning zebrafish *hspb8* focused on its expression at the mRNA level during the development of zebrafish embryos under normal and heat shock conditions [50,51]. Elicker et al. (2007) [50] show that the expression level of *hspb8^eefa1l1^* under normal conditions increases dramatically 12 h after fertilization. The increase is slower at 24 hpf (1 dpf), after which it decreases significantly at 2 dpf. The next stage they considered was 5 dpf, in which the expression level was assessed as slightly lower than that at 2dpf. Our research complements the results they obtained with data on the level of *hspb8* expression at 3 and 4 dpf under normal and heat shock conditions. We observed a smaller (about 2-fold) decrease in *hspb8* expression at 2 dpf, but on the third day the mRNA increased again to a level similar to that at 1 dpf and, as Elicker et al. observed, it gradually fell over the following days. The impact of heat shock on the *hspb8* expression is more evident in the case of our research (Figure 1A,B). It seems that the *hspb8* is strictly controlled during the early stages of zebrafish development, which may suggest its important role in this process.

The increase in heat shock-induced *hspb8* expression that we observed at the mRNA level had no effect on protein distribution within the zebrafish embryo (Figure 1C,D). As we revealed by our immunofluorescence staining analysis, in developing zebrafish embryos, Hspb8 is mostly expressed in muscle cells (both slow and fast fibers) in the proximity of Z and M lines and nervous tissues within the cytoplasm of neurons in the lateral spinal cord (Figure 2).

Next, we decided to supplement the characteristics of zebrafish Hspb8 with information on its binding partners. Through a co-IP assay, we detected plausibly general and tissue-specific interactions between Hspb8 and different proteins (Figure 3). According to results obtained in this study, zebrafish Hspb8 forms autophagy-inducing complexes with Bag3 and Hsc70. As an outcome of our analysis, other proteins involved in the formation of the autophagy-inducing complex such as dynein and 14-3-3 protein were also detected. Data on protein interactions obtained from different organisms also revealed the role of Hspb8 in the formation of complexes involved in this basic cellular process [9,11,12,13]. Together with our data, this implies the evolutionarily conserved function of Hspb8 in autophagy. Additionally, using the co-IP assay and WB analysis we detected an interaction between zebrafish Bag3 and LC3-I/-II, which is a central protein in autophagosome biogenesis. We also confirmed that in zebrafish, Hspb1 can interact with its protein family member Hspb8 [2] and Bag3, which is recognized as a scaffolding factor bringing together sHSPs and Hsp70s [15] (Figure 3). 

Proper functioning of the Hspb8/Bag3 complex is known to be crucial for skeletal muscle maintenance [14]. Since we detected interactions between zebrafish Hspb8 and Bag3 in zebrafish larvae, and confirmed their presence in the muscle tissue of embryos and adult individuals, it seems that in zebrafish this protein performs an evolutionarily conservative function consisting of participation in development and maintenance of muscle structure.

Through the co-IP assay, we also identified a potential interaction between Hspb8 and neuronal proteins Dd×20 (a partner of SMN, survival of motor neuron) and synaptotagmin, which suggests Hspb8′s involvement in neuronal cell functioning.

The interaction between Hspb8 and RNA helicase Dd×20 (DEAD box protein Dd×20, gemin3, DP103), which itself binds to the SMN protein, has so far been identified in mammalian cells [21,22]. Dd×20 can bind various partners, e.g., transcription factors [54]. Moreover, this protein seems to be necessary during the early stages of embryogenesis [23]. The interaction between Dd×20 and SMN is important for assembly and pre-mRNA processing [21,22]. Since the motor neuropathy-associated mutant Hspb8 form reveals increased interaction with Dd×20, it seems plausible that mutations influencing the structure and/or biochemical properties of Hspb8 can lead to modulation of functions executed by Dd×20 activity [21]. This seems particularly interesting in the context of the use of zebrafish as a model to study these kinds of human diseases.

Spatial-temporal expression of Hspb8 during zebrafish development suggests its involvement in muscle and nervous system maturation and functioning; therefore, we decided to test whether zebrafish Hspb8 is important during zebrafish development. Our analysis comprising morpholino-mediated *hspb8* knockdown was mostly focused on muscle analysis. Our experiment showed that the reduction of *hspb8* expression leads to the development of an abrupt phenotype that includes abnormalities in morphology such as curved body, altered tail region, and pericardial edema (Figure 4). Morphants (individuals with a reduced level of *hspb8* expression) exhibited changes in muscle structure, which were visible in polarized light as reduced birefringence in comparison to that of normal individuals. At the ultrastructural level, depletion of Hspb8 was manifested in the disruption of sarcomere organization seen as gaps between filaments within the sarcomere and the accumulation of glycogen granules in morphants’ muscles. Moreover, we also detected in morphants’ muscles the presence of structures such as membrane-bound autophagosomes containing glycogen, a mitochondrion with mitochondrial vacuolization, a swollen mitochondrion, and a structure built of a whorl-like inner membrane (Figure 4D). Our observations suggest that Hspb8 may be involved in muscle development and/or the maintenance of muscle structure in zebrafish. Of note, the presence of abrupt mitochondria seems to be interesting since the involvement of Hspb8 in the maintenance of the mitochondrial membrane potential and oxidative phosphorylation in mitochondria has also been confirmed [19,20]. Moreover, in *hspb8* KO (knock-out) model mice, myofibers showed accumulation of abnormally patterned mitochondria [35].

Further analysis of morpholino-mediated *hspb8* knockdown involved the assessment of zebrafish swimming abilities via a touch-evoked escape response assay (TER) (Figure 5). The TER assay demonstrated shorter swimming distance, as well as an abnormal trajectory of morphants in comparison to that of control individuals. Control group embryos exhibited a rectilinear type of movement characteristic of this age; morpholino-injected embryos moved by turning around their axis in a sinusoidal manner. These results indicate that the knockdown of *hspb8* caused general locomotion impairment in zebrafish embryos. 

It is well known that muscles and motor neurons are interdependent, and they form a coherent and functional system. This means that abnormalities such as denervation of one of the elements of the system they create may affect the functioning of the other [55]. Motor tests such as assessment of swimming behavior give us indirect information about the nervous system. However, it is difficult to prove the impact of knockdown of *hspb8* on the development/functioning of the nervous system. To determine this, more precise analysis and tools such as genome editing are required.

The recently generated transgenic mouse models of mutant hspb8 could be possibly useful in the hspb8 functional studies, however, they show divergent phenotypes [31,32,33,34,35]. For example, some *hspb8* KO model mice [32] exhibited normal behavior and physiology, but they showed increased susceptibility to heart failure under the specific context of cardiac overload, whereas others did not develop a strong neuropathic or myopathic phenotype [35]. However, in the mentioned case, *hspb8* KO myofibers showed accumulation of abnormally patterned mitochondria [35]. Cardiac-specific Hspb8 mutant (K141N) transgenic mice exhibited mild hypertrophy and apical fibrosis with slightly reduced cardiac function [33]. Other KI (knock-in) lines, intended to mimic human neuropathic and myopathic diseases triggered due to the presence of the K141N missense mutation, developed a variety of symptoms such as loss of myelinated axons, accumulation of mutant Hspb8, and reduction of autophagy markers. They also showed a progressive myofibrillar myopathy followed by Z-disk disorganization and decreased locomotor activity [35]. 

Research on *hspb8* function with the consideration of the cardiac aspect seems to be very important. In the case of our study, since we observed changes in the heart area in morphants during our morpholino-mediated knockdown experiments, we can assume that Hspb8 may play a vital role in heart functioning. However, further analyses should be undertaken to deduce whether and possibly how the *hspb8* knockdown in zebrafish affects heart functioning.

As could be expected, different spectra of symptoms revealed by different models of mutant hspb8 are closely associated with the mutation type. It seems that in the case of missense *hspb8* mutations, the toxic effect is launched primarily due to the tendency of mutant Hspb8 to accumulate and form large aggregates [19,35]. On the other hand, frame-shift mutations of the gene coding for Hspb8, which leads to the development of a myopathic phenotype, trigger a loss-of-function mechanism [8]. One should be aware that because Hspb8 acts in complexes with other chaperones and partners, the consequences of the effects of the reduction of Hspb8 expression are difficult to predict.

Notably, many studies on the depletion of other sHSPs have been carried out. For example, in the case of Hspb1, one of the Hspb8 partners that we confirmed in this study, its depletion in the mouse model caused myofiber defects [56]. In addition, in a zebrafish model, inhibition of Hspb1 expression allowed for confirmation of its role in craniofacial muscle development [57]. The generation of a stable zebrafish *hspb8* knockout line seems very tempting and also promising in the context of results presented in this study, especially since the results obtained so far using various mouse models of mutant *hsp8*, although undoubtedly valuable, still do not give us a full picture of the Hspb8 function. 

## 5. Conclusions

In this study, we presented a detailed characterization of zebrafish Hspb8. We confirmed and broadened the knowledge of its expression pattern during development under normal and heat shock conditions as well as its tissue- and subcellular-specific localization. The analysis of protein partners allowed us to conclude that, similarly to other organisms, zebrafish Hspb8 can be involved in the formation of the autophagy-inducing complex through interactions with Bag3 and other autophagy-inducing proteins such as Hsc70, dynein, and 14-3-3. It can also interact with its protein family member Hspb1. Moreover, identification of the possibility of interaction between Hspb8 and Dd×20 and synaptotagmin suggests its involvement in neuronal cell functioning. Our morpholino-mediated *hspb8* knockdown experiments suggest that Hspb8 is involved in muscle development and/or the maintenance of muscle structure in zebrafish, and its depletion caused general locomotion impairment in zebrafish embryos. Our research provides a valuable resource for the potential use of the zebrafish as a model for studying pathological conditions associated with Hspb8 disorders.

Future studies using genome editing tools such as CRISPR/Cas9-mediated knockout are needed to understand the exact role of Hspb8 during zebrafish development as well as adult organism functioning. The generation of a stable zebrafish line enables long-term observation of the effects of *hspb8* knockout and will eliminate the typical limitations of the morpholino-based method. Moreover, in addition to the toxic gain-of-function mechanism for Hspb8 is associated with human pathological conditions that has been confirmed by various researchers, the loss-of-function mechanism may cause the development of related pathological conditions. Studies using non-mouse models may contribute to a better understanding of the background of various diseases associated with the functional impairment of the CASA elements. From a broader perspective, the generation of zebrafish models via genome editing tools should allow development of a therapeutic strategy in motor neuron and muscle disease associated with mutations of the gene encoding the Hspb8 human counterpart. 

## Figures and Tables

**Table 1:** The sequences of gene-specific primers used in the real-time quantitative PCR analyses.

Target Gene	Seq F (Forward Primer)	Seq R (Reverse Primer)
*Danio rerio* ribosomal protein L13a (rpl13a)	CGCTATTGTGGCCAAGCAAG	TCTTGCGGAGGAAAGCCAAA
*Danio rerio* actin, beta 1 (actb1)	CGAGCTGTCTTCCCATCCA	TCACCAACGTAGCTGTCTTTCTG
*Danio rerio* eukaryotic translation elongation factor 1 alpha 1, like 1 (eef1a1l1)	CTGGAGGCCAGCTCAAACAT	ATCAAGAAGAGTAGTACCGCTAGCATTAC
*hspb8*	CAGCATGACTTCAACCACAAC	CACGGGCTTGGAACAAATAAG

**Table 2:** The sequences of morpholino.

Name	Sequence	Concentration
Morpholino 1 (M1)	5ʹ-TATAATAATCCCCCTCTGCCATTGT-3ʹ	0.2 mM
Morpholino 2 (M2)	5ʹ-AAACTCTGGATAAAGTGTGTTTGGC-3ʹ	0.3 mM

## Supplementary Materials

The following are available online at https://www.mdpi.com/2073-4409/9/6/1562/s1, Figure S1: Control of morpholino-mediated *hspb8* knockdown effectiveness in 72 hpf zebrafish embryos. Figure S2: Control of the specificity of a commercially available monoclonal antibody raised against human Hspb8 used in this study for the detection of zebrafish Hspb8.

## Abbreviations

Bag3	Bcl2-associated athanogene 3
BF	birefringence
*β-actin*	gene of beta *actin*
CASA	chaperone-assisted selective autophagy
CHIP	carboxyl terminus of HSC70-interacting protein
CMT2L	Charcot-Marie-Tooth disease type 2L
co-IP	co-immunoprecipitation
Dd×20	DEAD box protein
dHMN	distal hereditary motor neuropathy (type IIA)
dpf	days post fertilization
*eef1a1l1*	gene of *Danio rerio* eukaryotic translation elongation factor 1 alpha 1, like 1
fps	frames per second
hpf	hours post fertilization
Hsp70	heat shock protein 70
Hspb	heat shock protein B
*hspb8 ^β-actin^*	mRNA of *Hspb8* normalized to mRNA of *beta actin*
*hspb8^eef1a1l1^*	mRNA of *Hspb8* normalized to mRNA of *eef1a1l1*
*hspb8 ^rpl13a^*	mRNA of *Hspb8* normalized to mRNA of *rpl13a*
KI	knock-in
KO	knock-out
LC-MS	liquid chromatography-mass spectrometry
MO	morpholino oligonucleotides
PBS	phosphate-buffered saline
PBST	phosphate-buffered saline with 1% (v/v) Tween-20
PFA	paraformaldehyde
*rpl13a*	gene of *Danio rerio* ribosomal protein L13a
RT qPCR	real-time quantitative polymerase chain reaction
RVM	rimmed vacuolar myopathy
sHSP	small heat shock protein
SMN	survival of motor neuron
TER	touch-evoked response
WB	Western blot
WT	wild type
