# Supplementary material for: Characterization of Hspb8 in Zebrafish
Source: Cells. 2020 Jun 26;9(6):1562. doi: 10.3390/cells9061562 (PMC7348923; doi:10.3390/cells9061562)
Supplement: Supplementary file 1 [file cells-09-01562-s001.pdf]

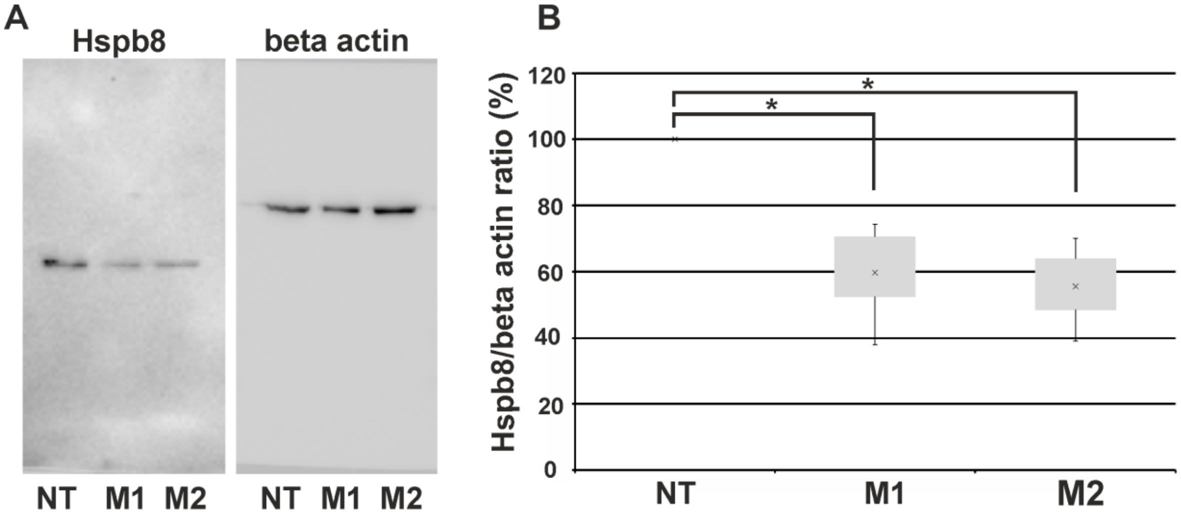

Figure S1. Control of morpholino-mediated *hspb8* knockdown effectiveness in 72 hpf zebrafish embryos. (A) The representative Western blot analysis of Hspb8 level, with β-actin used as a loading control. (B) The graph summarizes the Hspb8/β-actin ratio. The Hspb8 quantity in the non-treated (NT) group was taken as 100%. The experiment was performed 3 times, n = 10. Asterisks (*) indicate significant differences, $p>0.05$. Error bars show the standard deviation. NT – non-treated embryos, M1, and M2 – morphant groups.

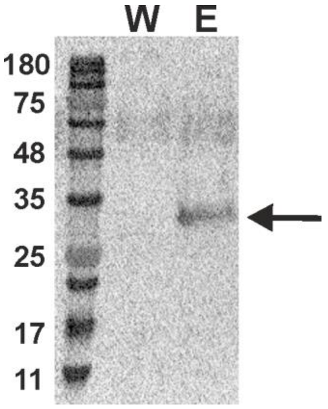

Figure S2: Control of the specificity of a commercially available monoclonal antibody raised against human Hspb8 used in this study for the detection of zebrafish Hspb8
